# Targeting FGFR4 Inhibits Hepatocellular Carcinoma in Preclinical Mouse Models

**DOI:** 10.1371/journal.pone.0036713

**Published:** 2012-05-15

**Authors:** Dorothy M. French, Benjamin C. Lin, Manping Wang, Camellia Adams, Theresa Shek, Kathy Hötzel, Brad Bolon, Ronald Ferrando, Craig Blackmore, Kurt Schroeder, Luis A. Rodriguez, Maria Hristopoulos, Rayna Venook, Avi Ashkenazi, Luc R. Desnoyers

**Affiliations:** 1 Department of Pathology, Genentech, Inc., South San Francisco, California, United States of America; 2 Department of Molecular Oncology, Genentech, Inc., South San Francisco, California, United States of America; 3 Department of Antibody Engineering, Genentech, Inc., South San Francisco, California, United States of America; 4 Department of Cancer Signaling and Translational Oncology, Genentech, Inc., South San Francisco, California, United States of America; 5 GEMpath, Inc., Longmont, Colorado, United States of America; University of Michigan School of Medicine, United States of America

## Abstract

The fibroblast growth factor (FGF)-FGF receptor (FGFR) signaling system plays critical roles in a variety of normal developmental and physiological processes. It is also well documented that dysregulation of FGF-FGFR signaling may have important roles in tumor development and progression. The FGFR4–FGF19 signaling axis has been implicated in the development of hepatocellular carcinomas (HCCs) in mice, and potentially in humans. In this study, we demonstrate that FGFR4 is required for hepatocarcinogenesis; the progeny of *FGF19* transgenic mice, which have previously been shown to develop HCCs, bred with *FGFR4* knockout mice fail to develop liver tumors. To further test the importance of FGFR4 in HCC, we developed a blocking anti-FGFR4 monoclonal antibody (LD1). LD1 inhibited: 1) FGF1 and FGF19 binding to FGFR4, 2) FGFR4–mediated signaling, colony formation, and proliferation in vitro, and 3) tumor growth in a preclinical model of liver cancer in vivo. Finally, we show that FGFR4 expression is elevated in several types of cancer, including liver cancer, as compared to normal tissues. These findings suggest a modulatory role for FGFR4 in the development and progression of hepatocellular carcinoma and that FGFR4 may be an important and novel therapeutic target in treating this disease.

## Introduction

Fibroblast growth factors (FGFs) comprise a family of 22 structurally related polypeptides with diverse biological activities [1]. Most of these signaling molecules function by binding to and activating members of the FGF receptor (FGFR) family of receptor tyrosine kinases, of which there are four members designated FGFR1–4 [2]. These receptor-ligand interactions result in receptor dimerization and autophosphorylation, formation of complexes with membrane-associated and cytosolic accessory proteins, and initiation of multiple signaling cascades [3]. The FGFR-FGF signaling system plays important roles in development and tissue repair by regulating cellular functions/processes such as growth, differentiation, migration, morphogenesis, and angiogenesis. Not surprisingly, dysregulation of this signaling axis has also been shown to play significant roles in tumor development and progression.

Alterations in FGFRs (i.e. overexpression, mutation, translocation, and truncation) are associated with a number of human cancers, including myeloma, breast, stomach, colon, bladder, pancreatic, and hepatocellular carcinomas 4,5,6,7,8,9,10,11,12,13. Hepatocellular carcinoma (HCC) is one of the leading global causes of cancer related deaths, resulting in over half a million fatalities per year [14]. While the role of FGFR4 in cancer remains to be fully elucidated, several findings suggest that this receptor may be an important player in HCC development and/or progression. FGFR4 is the predominant FGFR isoform present in human hepatocytes [15]. We have also previously reported that liver tissue has the highest transcript levels of *FGFR4* [16]. In addition to FGFR4 being overexpressed in HCCs, several missense genetic alterations have been observed in HCC patient samples [17]. Notably, a highly frequent G388R single nucleotide polymorphism in FGFR4 (associated with reduced survival for head and neck carcinoma, as well as a more aggressive phenotype for colon, soft tissue, prostate, and breast carcinomas) was identified [17]. Furthermore, it has been previously demonstrated that ectopic expression of FGF19 (i.e. FGFR4-specific ligand) in mice promotes hepatocyte proliferation, hepatocellular dysplasia, and neoplasia [18]. We and others have also recently demonstrated that Klotho β (KLB) is required for the liver-specific activities of FGF19 and that KLB is most highly expressed in liver, along with FGFR4, further supporting the premise that the liver uniquely possesses the necessary machinery required for the activity of this signaling system [16], [19]. Finally, it has been reported that FGFR4-FGF19 can crosstalk with β-catenin signaling and that inactivation of either FGFR4 or FGF19 reduces tumorigenesis [20].

To test the importance of FGFR4 in hepatocellular carcinoma, we evaluated the effect of its ablation in a genetically engineered mouse model of HCC and assessed the effects of therapeutic FGFR4 neutralization in relevant mouse tumor models. We demonstrate here that FGFR4 is required for FGF19-mediated liver tumorigenesis in vivo and show that treatment with an FGFR4 neutralizing antibody inhibited FGFR4-mediated signaling, proliferation, and colony formation in cell-based assays and tumor growth in preclinical models of HCC in vivo. We also show that FGFR4 expression is elevated in several types of cancer, including liver cancer, as compared to normal tissues. These findings provide evidence for a modulatory role of FGFR4 in HCC development and progression and suggest that FGFR4 may be an important and novel therapeutic target in treating this disease.

## Results

### FGFR4 is Required for Hepatocarcinogenesis in FGF19 Transgenic Mice

**Figure 1:**
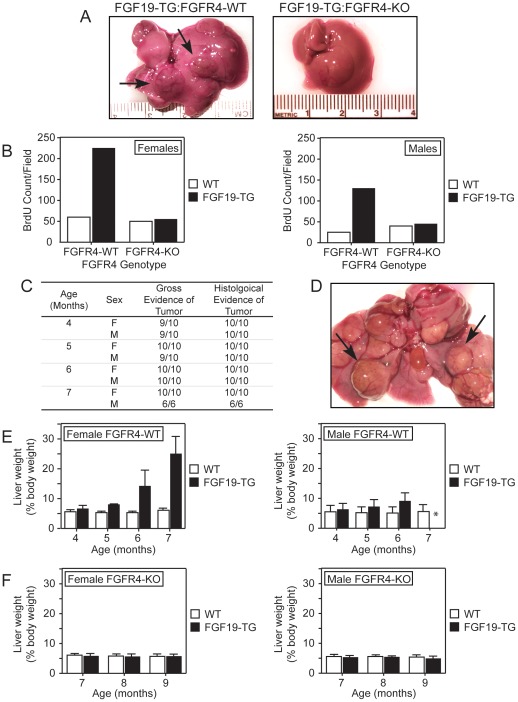
FGFR4 is required for FGF19-mediated liver tumorigenesis. *A*, Multiple, large, raised tumors (arrows) protruding from the hepatic surface of a 10-month-old FGF19-TG:FGFR4-WT mouse (left panel). Liver from a 10-month-old FGF19-TG:FGFR4-KO mouse (right panel). *B*, BrdU incorporation in female (left panel) and male (right panel) FGF19-TG or wild type mice bred with FGFR4-KO or FGFR4-WT mice. *C*, Prevalence of liver tumors in male and female FGF19-TG mice treated with DEN as determined by gross and histological examinations. *D*, Multiple, large, raised tumors (arrows) on the surface of the liver of a 4-month-old FGF19-TG:FGFR4-WT mouse treated with DEN. *E*, Liver weights from FGF19-TG or wild type female (left panel) and male (right panel) mice treated with DEN. The asterisk (*) indicates that the weight of the liver could not be measured from the 7-month time point for male FGF19-TG mice treated with DEN because none survived past 6 months of age. *F*, Liver weights of FGF19-TG or wild type female (left panel) and male (right panel) FGFR4-KO mice treated with DEN.

The exogenous expression of FGF19 in transgenic mice was previously shown to cause HCC by the age of 10 months [18]. To assess whether FGFR4 is involved in this FGF19-mediated tumorigenesis we bred the *FGF19* transgenic (FGF19-TG) mice with *FGFR4* knockout (FGFR4-KO) mice or *FGFR4* wild type (FGFR4-WT) mice. The mice were necropsied at various time points and liver carcinogenesis was assessed by performing gross and pathological histology examinations and by measuring preneoplastic hepatocellular proliferation (i.e. BrdU incorporation). The development of HCC in FGF19-TG:FGFR4-WT mice was as previously described [18]. Contrary to the FGF19-TG:FGFR4-WT mice, the FGF19-TG:FGFR4-KO mice did not develop gross or histological evidence of hepatocellular neoplasia at any time during this experiment (Fig. 1A). Also, preneoplastic hepatocellular proliferation was significantly elevated in FGF19-TG mice that had the FGFR4-WT genotype, but was not evident in the FGF19-TG:FGFR4-KO littermates (Fig. 1B). Consistent with the previously reported higher frequency and severity of tumor development in female FGF19-TG mice [18], the BrdU incorporation was increased in FGF19-TG:FGFR4-WT females as compared to the corresponding males (compare left and right panels of Fig. 1B). We also evaluated the effect of diethylnitrosamine (DEN), a potent liver carcinogen, on the development of HCC in FGF19-TG mice. The administration of DEN accelerated the development of HCC in FGF19-TG:FGFR4-WT mice. The entire range of preneoplastic and neoplastic lesions – altered (basophilic) hepatic foci, pericentral hepatocyte dysplasia, well differentiated hepatocellular neoplasms, and aggressive hepatocellular carcinomas – was seen in livers from all DEN-treated FGF19-TG:FGFR4-WT animals by 4 months of age (Fig. 1C) as compared to 10 months of age for the non-DEN-treated FGF19-TG:FGFR4-WT mice. The cardinal morphologic characteristic of livers from almost all FGF19-TG:FGFR4-WT mice at all time points was grossly evident nodules of HCC on multiple lobes (Fig. 1D). The tumor burden was evaluated by measuring liver weight. The relative liver weights increased progressively at all time points in FGF19-TG:FGFR4-WT mice treated with DEN (Fig. 1E). Interestingly, the increase in liver weight was more pronounced in females (2.7-fold at 6 months) than in males (1.8-fold at 6 months) (compare left and right panels of Fig. 1E). It should be noted that none of the males survived past 6 months of age (Fig. 1E). The hepatocarcinogenesis observed in the FGF19-TG:FGFR4-WT mice treated with DEN was abolished by the removal of FGFR4 expression in the FGFR4-KO mice. Accordingly, the relative liver weight of FGF19-TG:FGFR4-KO mice remained constant during adulthood (Fig. 1F). These results suggest that FGFR4 expression is required for FGF19-promoted hepatocarcinogenesis in mice.

### Generation of an Anti-FGFR4 Neutralizing Monoclonal Antibody

**Figure 2:**
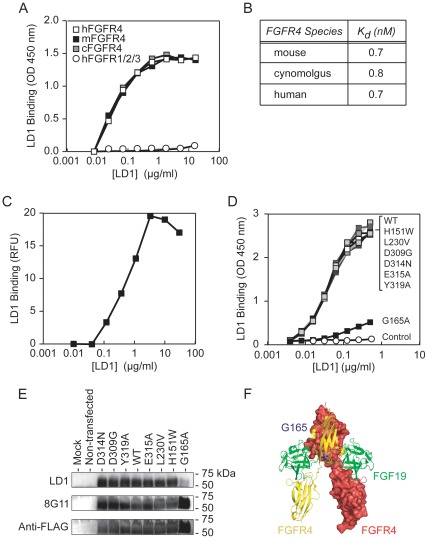
LD1 binds to FGFR4. *A*, LD1 binds to human (h), mouse (m), and cynomolgus monkey (c) FGFR4, but does not bind to hFGFR1, hFGFR2, or hFGFR3. The binding of LD1 to immobilized FGFR-Fc chimeric proteins was determined by solid phase binding assay. *B*, Affinity of LD1 binding to mouse, cynomolgus monkey, and human FGFR4 as determined by surface plasmon resonance. *C*, Binding of LD1 to hFGFR4 expressed at the cell surface of stably transfected HEK293 cells as measured by FACS (RFU  =  Relative Fluorescence Unit). *D*, The binding of LD1 to immobilized hFGFR4-Flag chimeric proteins bearing point mutations as measured by a solid phase binding assay. *E*, The binding of LD1 to hFGFR4-Flag chimeric proteins bearing point mutations as evaluated by Western blot. Mutated proteins were electrophoresed and sequentially immunoblotted using LD1, an anti-FGFR4 (8G11), and an anti-Flag antibody. *F*, Dimer model illustrating the position of G165 (blue) on FGFR4 (red and yellow) bound to FGF19 (green).

To evaluate whether targeting FGFR4 could have a therapeutic impact in HCC we generated an FGFR4-specific monoclonal antibody by immunizing FGFR4-KO mice with recombinant mouse and human FGFR4. One of the resulting clones, designated as LD1, was selected for the specificity of its binding to mouse, cynomolgus monkey, and human FGFR4 (Figs. 2A and S1). This antibody did not bind to mouse or human FGFR1, FGFR2, or FGFR3 (Figs. 2A and S1). Surface plasmon resonance analysis revealed that LD1 bound to mouse, cynomolgus monkey, and human FGFR4 with comparable affinity (Fig. 2B). We used flow cytometry to evaluate whether LD1 bound to FGFR4 present at the cell surface. The specific binding of LD1 to HEK293 cells stably transfected with human *FGFR4* was proportional to the concentration of antibody added (Fig. 2C). There was no binding of LD1 to control HEK293 cells stably transfected with an empty vector (Fig. S1). Together these data demonstrate that LD1 binds specifically to mouse, cynomolgus monkey, and human FGFR4 and also recognizes the human receptor when expressed at the cell surface.

To map the FGFR4 epitope for LD1, we compared the amino acid sequences of mouse and human FGFR1, FGFR2, FGFR3, and FGFR4. Eight amino acids were selected based on their similarity between the FGFR4 orthologs and their dissimilarity in the FGFR1-3 orthologs. These amino acids in FGFR4 were substituted with the amino acids present at the equivalent positions in FGFR3 to generate eight different mutant constructs of human FGFR4. These constructs were expressed and evaluated for LD1 binding using a solid phase binding assay. LD1 bound equally well to wild type FGFR4 and most of the mutant constructs; G165A was the only FGFR4 mutant for which LD1 binding was compromised (Fig. 2D). LD1 did not bind to the negative control wild type FGFR3 (Fig. 2D). We also tested the binding of LD1 to the mutant constructs using immunoblot analysis. All previously described protein constructs were reduced, denatured, electrophoresed, and electro-transferred to nitrocellulose. The nitrocellulose membrane was sequentially incubated with LD1, an anti-FGFR4 antibody recognizing a different epitope (8G11), or an anti-FLAG antibody. The anti-FLAG antibody and 8G11 detected wild type FGFR4 and all FGFR4 mutant constructs while LD1 detected all constructs equally well with the exception of the G165A mutant (Fig. 2E). No protein band was detected by any of the antibodies in the control lanes (Fig. 2E). We generated a three-dimensional model of an FGFR4 dimer bound to two molecules of FGF19 to visualize the location of G165 (Fig. 2F). G165 is localized in the center of the FGFR4-FGF19 complex at the point of contact between the two FGFR4 units. Together these results show that G165 is critical for the interaction of LD1 with human FGFR4. The binding of LD1 to reduced and denatured FGFR4 also suggests that the epitope does not depend on ternary confirmation.

**Figure 3:**
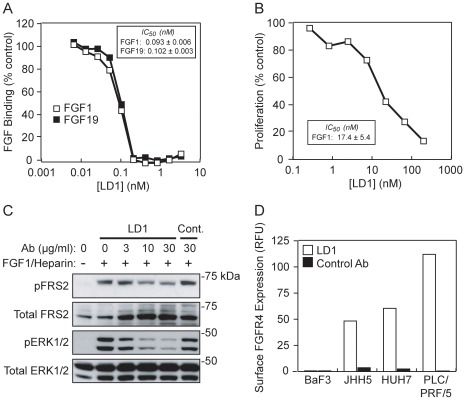
LD1 inhibits FGFR4 activities. *A*, LD1 inhibits FGFR4 binding to FGF1 and FGF19 as determined by solid phase binding assay. *B*, LD1 inhibits FGF1-stimulated proliferation of BaF3 cells stably expressing FGFR4/R1. *C*, LD1 inhibits FGFR4 signaling in L6 cells stably expressing FGFR4. *D*, Cell surface expression of FGFR4 protein in a subset of liver tumor cell lines as determined by FACS analysis using LD1.

Next we tested whether LD1 could block the binding of FGF1 and FGF19 to FGFR4 using a solid phase receptor-binding assay. The LD1 inhibition of FGF binding was dose-dependent and reached an IC_50_ of 0.093±0.006 nM for FGF1 and 0.102±0.003 nM for FGF19 (Fig. 3A). To evaluate whether LD1 could inhibit the functions of FGFR4 expressed at the cell surface we first utilized a BaF3 murine pro-B cell line stably transfected with a chimeric construct that encodes for the extracellular domain of FGFR4 and the intracellular domain of FGFR1 (BaF3/FGFR4/R1). The wild type BaF3 cell line is an interleukin-3 (IL-3)-dependent cell line that does not express any FGFRs. BaF3 cells transfected with *FGFR* expression constructs proliferate in the absence of IL-3 when stimulated with FGF and heparin [21]. The transfection of this construct allowed us to substitute FGFs for IL-3 to support the growth of the BaF3 cells. In the presence of 5 nM FGF1, LD1 inhibited the proliferation of BaF3/FGFR4/R1 cells with an IC_50_ of 17.4±5.4 nM (Fig. 3B).

We also used the L6 rat skeletal muscle cell line stably transfected with a vector expressing FGFR4 (L6/FGFR4) to evaluate the effect of LD1 on FGF signaling. The addition of FGF1 and heparin to the L6/FGFR4 cell cultures activated the FGFR pathway as demonstrated by the phosphorylation of FGFR substrate 2 (FRS2) and extracellular signal-regulated kinase 1/2 (ERK1/2) while LD1 inhibited the ligand-induced phosphorylation of these secondary messengers in a dose-dependent manner (Fig. 3C). Interestingly, the addition of LD1 also triggered an increase in total FRS2 content in these cells (Fig. 3C).

Using flow cytometry we evaluated the binding of LD1 and confirmed the expression of FGFR4 at the cell surface of a subset of HCC cell lines. LD1 bound most highly to PLC/PRF/5 cells and bound to a lesser extent to HUH7 and JHH5 cells (Fig. 3D). The binding of a control antibody to the surface of these cells was negligible (Fig. 3D). Furthermore, the binding of LD1 and the control antibody to the surface of BaF3 cells, which were used as a negative control because they do not express FGFR4, was also negligible (Fig. 3D).

### LD1 Inhibits FGFR4 Functions in Liver Cancer Cell Lines

**Figure 4:**
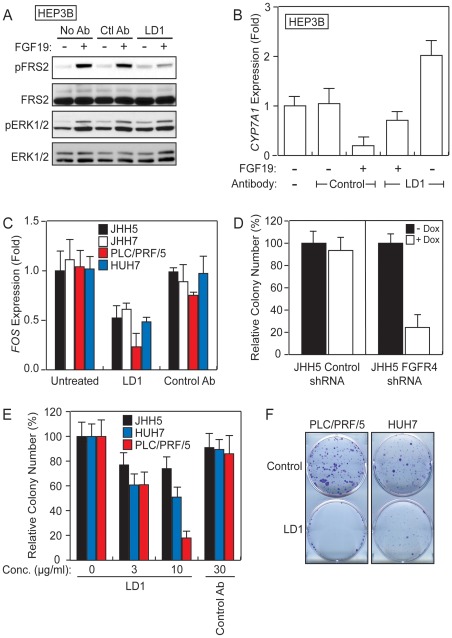
LD1 inhibits FGFR4 biological activities in liver cancer cell lines. *A*, LD1 inhibits FGFR4 signaling in HEP3B cells as evaluated by Western blot. *B*, LD1 inhibits the FGFR4-regulated *CYP7A1* repression in HEP3B cells. *CYP7A1* levels are represented as fold expression relative to the level in untreated cells. *C*, LD1 inhibits FGFR4-regulated *FOS* expression in a panel of liver cancer cell lines. The results are represented as fold expression relative to the *FOS* level in untreated cells. *D*, Inhibition of colony formation by repression of FGFR4 expression in JHH5 cells stably transfected with an FGFR4 shRNA doxycycline-inducible vector. *E*, Enumeration of LD1-inhibited liver cancer cell line colony formation. The values are represented as percent of the number of colonies enumerated in the absence of added LD1. *F*, LD1 inhibits HCC cell line colony formation.

The inhibitory activity of LD1 was characterized using liver cancer cell lines with various levels of endogenous FGFR (i.e. FGFR1-4) expression (Fig. S2). In HEP3B cells, the addition of FGF19 triggered the phosphorylation of FRS2 and ERK1/2 while LD1 inhibited the FGF19-stimulated phosphorylation of FRS2 (Fig. 4A), similar to its effect on L6/FGFR4 cells. LD1, however, did not appreciably alter the phosphorylation of ERK1/2 (Fig. 4A).

The expression of cholesterol 7α-hydroxylase (*CYP7A1*) and c-Fos (*FOS*) genes is modulated by FGF19 in liver cell lines [16], [22]. We tested whether LD1 could inhibit this FGF19-mediated gene modulation. In HEP3B cells, the addition of FGF19 reduced the expression of *CYP7A1* by 81% (Fig. 4B). The addition of LD1 restored 67% of the basal expression of *CYP7A1* (Fig. 4B). In the absence of added FGF19, LD1 increased *CYP7A1* expression by 2-fold (Fig. 4B). Although the addition of FGF19 did not affect the expression of *CYP7A1* in HUH7 cells, the addition of LD1 had a similar effect as in HEP3B cells, increasing the expression of this gene by 2.9- and 3.5-fold in the presence or the absence of FGF19, respectively (Fig. S3). The addition of a negative control antibody had no effect on the expression of *CYP7A1* in either HEP3B or HUH7 cells (Figs. 4B and S3, respectively). Interestingly, the addition of LD1 leads to the upregulation of *CYP7A1* expression in the absence of exogenously added FGFR4 ligand in both HEP3B and HUH7 cells. This indicates that LD1 inhibits the FGFR4 basal activity possibly maintained by an FGFR4 ligand autocrine/paracrine loop.

To further evaluate the effect of LD1 on the basal activity of FGFR4 we measured the expression of *FOS* in the absence of exogenously added FGFR4 ligand. The activation of the FGFR4 pathway was previously shown to increase *FOS* expression [16]. The addition of LD1 decreased the basal expression of *FOS* by 50% in JHH5, JHH7, and HUH7 cell lines and by 75% in the PLC/PRF/5 cell line; addition of a control antibody had no effect on basal *FOS* expression (Fig. 4C). These results demonstrate the ability of LD1 to inhibit the basal activity of FGFR4.

### LD1 Inhibits Colony Formation

We first measured colony formation by JHH5 cells stably transfected with a doxycycline-inducible FGFR4-specific shRNA or a control shRNA. Although there was no difference in the ability of JHH5 cells transfected with the control construct to form colonies in the absence or presence of doxycycline, the addition of doxycycline to the JHH5 cells transfected with the FGFR4 shRNA construct inhibited colony formation by 76% as compared to cells in the absence of doxycycline (Fig. 4D). This result suggests that FGFR4 is involved in the colony formation of liver cancer cell lines.

Next we tested the ability of LD1 to inhibit colony formation by a panel of liver cancer cell lines. The addition of LD1 to cultures of JHH5, HUH7, and PLC/PRF/5 cells caused a dose-dependent reduction in colony formation, reaching a maximum inhibition of 26%, 50%, and 82%, respectively (Fig. 4E). Representative examples of PLC/PRF/5 and HUH7 cell cultures are shown in Fig. 4F. The addition of a control antibody did not affect colony formation (Figs. 4E and 4F). These results indicate that LD1 inhibits FGFR4-mediated colony formation in liver cancer cell lines.

### LD1 Inhibits FGFR4 in vivo Activity

**Figure 5:**
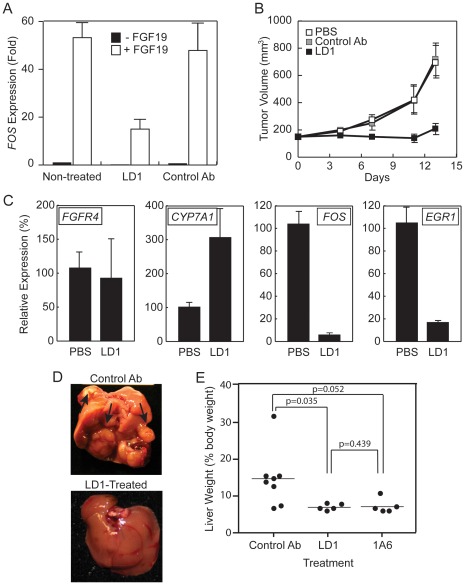
In vivo efficacy of LD1. *A*, LD1 inhibits FGF19-regulated *FOS* expression in mouse liver. The results are represented as fold expression relative to *FOS* levels in the livers of non-treated mice. *B*, LD1 (30 mg/kg; once weekly) inhibits HUH7 xenograft tumor growth in vivo. *C*, Effects of LD1 on the mRNA expression of *FGFR4*, *CYP7A1*, *FOS*, and *EGR1* in HUH7 xenograft tumors from Fig. 5B. *D*, Multiple, large, raised tumors (arrows) protruding from the hepatic surface of a DEN-accelerated FGF19-TG:FGFR4-WT mouse treated with a control antibody (upper panel). Liver of DEN-accelerated FGF19-TG:FGFR4-WT mouse treated with LD1 (lower panel). *E*, Liver weights of DEN–accelerated FGF19-TG:FGFR4-WT mice treated with control antibody, LD1, or 1A6 (anti-FGF19 antibody).

We evaluated the in vivo efficacy of LD1 by measuring the FGF19-triggered *FOS* induction in the livers of mice injected with LD1 or a control antibody. We chose to monitor the *FOS* response to FGF19 because *FOS* induction in the liver is sensitive to FGF19 stimulation [16]. *FOS* expression was 53-fold higher in the livers of mice treated with FGF19 compared with livers of mice treated with phosphate buffered saline (PBS) (Fig. 5A). The administration of LD1 48 hours prior to the injection of FGF19 reduced the *FOS* induction by 3.5-fold (Fig. 5A). LD1 also reduced the basal level of *FOS* expression in naïve mice by 6-fold (Fig. 5A). The injection of a control antibody did not alter the basal or the FGF19-stimulated expression of *FOS* compared to the non-treated mice (Fig. 5A). These data demonstrate the in vivo efficacy of LD1 at inhibiting the basal and the FGF19-stimulated FGFR4 activity.

### LD1 inhibits tumor growth in vivo

To examine the in vivo efficacy of LD1 at inhibiting tumor growth we first utilized the HUH7 liver cancer cell line xenograft model. Mice bearing established tumors (approximately 150 mm^3^) were dosed weekly with 30 mg/kg LD1, 30 mg/kg control antibody, or PBS. After 13 days, the HUH7 tumors of mice treated with either PBS or control antibody grew to an average size of 720 mm^3^ (Fig. 5B). However, the HUH7 tumors of mice treated with LD1 grew to an average size of 28 mm^3^, a 96% inhibition of tumor growth as compared to control antibody or PBS (Fig. 5B). In a repeat experiment, the administration of 30 mg/kg of LD1 twice per week caused complete tumor growth inhibition (Fig. S4). At necropsy, the tumors were excised and the effect of LD1 on the expression of *FGFR4* and FGFR4-regulated genes was evaluated. The administration of LD1 did not affect *FGFR4* expression in HUH7 xenograft tumors (Fig. 5C). However, LD1 increased the expression of *CYP7A1* by 3-fold compared to the level of expression of *CYP7A1* measured in the tumors of PBS-treated mice (Fig. 5C). LD1 also reduced the expression of *FOS* and *EGR1* by 17- and 6-fold, respectively, compared to PBS-treated mice (Fig. 5C).

To further evaluate the in vivo efficacy of LD1 we used the FGF19-TG mouse model. FGF19-TG mice were treated with DEN at 15 days of age to accelerate tumorigenesis and then randomly grouped into 3 cohorts at 4 weeks of age. One group received a control antibody and the other two groups received either LD1 or an anti-FGF19 antibody (1A6) on a weekly basis. 1A6 was previously shown to prevent tumor formation in FGF19-TG mice [23]. After 6 months, the mice were necropsied and the livers were excised for analyses. The livers of mice treated with the control antibody had grossly evident large nodules on multiple lobes (Fig. 5D). However, the livers of mice treated with LD1 (Fig. 5D) or 1A6 (data not shown) had no evidence of neoplasia. We also measured liver weights to evaluate tumor burden because this parameter was previously shown to strongly correlate with percent tumor volume in the FGF19-TG model [18], [23]. The weight of the livers from mice treated with LD1 or 1A6 was significantly (p = 0.035 and p = 0.052, respectively) lower than the weight of the livers from mice treated with control antibody (Fig. 5E). The difference in liver weight between mice treated with LD1 and the mice treated with 1A6 was not significant (p = 0.439) (Fig. 5E). Together these data clearly demonstrate the in vivo efficacy of LD1 at inhibiting hepatocellular carcinoma in preclinical models.

### FGFR4 Expression is Altered in Cancer

**Figure 6:**
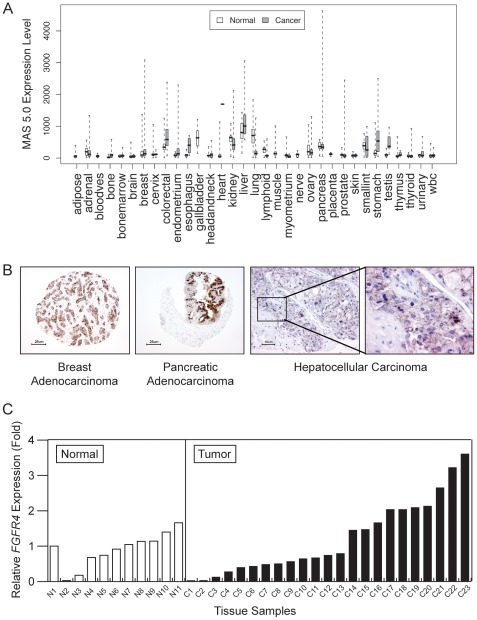
FGFR4 expression is deregulated in cancer. *A*, Whisker-box plots show *FGFR4* expression in human tumors and normal tissues as determined by mRNA analysis of the BioExpress database. The *center line* indicates the median; the *box* represents the interquartile range between the first and third quartiles. “Whiskers” extend from the interquartile to the positions of extreme values. B, FGFR4 immunostaining in samples of breast (×100 magnification) and pancreatic (×100 magnification) adenocarcinomas, and hepatocellular carcinoma (×200 magnification and ×400 magnification). *C*, *FGFR4* mRNA expression in a panel of human normal liver and liver tumors as determined by qRT-PCR. The value for each sample is represented as fold expression relative to the level observed in sample N1.

We evaluated *FGFR4* expression in a variety of human normal and cancerous tissues by analyzing the BioExpress database (Gene Logic, Inc., Gaithersburg, MD, USA). *FGFR4* expression is highly variable in most types of cancer. Compared to normal tissues, *FGFR4* expression was elevated in liver, colorectal, stomach, esophageal, and testicular cancers, but diminished in kidney, lung, lymphoid, and small intestine cancers (Fig. 6A). Using immunohistochemistry we localized FGFR4 in a panel of lung, breast, pancreas, and ovarian adenocarcinomas, lung squamous cell carcinoma, hepatocellular carcinoma, thyroid carcinoma, and normal lung, pancreas, and thyroid samples. The detection of FGFR4 gave rise to membranous and cytoplasmic staining in normal and neoplastic epithelial cells (representative examples are shown in Fig. 6B). Compared to normal tissues, higher grades of staining were generally found in tumor samples. Moderate to marked labeling by anti-FGFR4 was apparent in tumors from pancreas (in 41% of specimens), breast (46%), lung (31%), ovary (41%), colon (90%), liver (33%), and thyroid (11%) (Table S1 and ref. [23]). The widespread expression of FGFR4 in human HCC was also previously confirmed by *in situ* hybridization [23]. Because a link between FGFR4 and HCC has already been suggested we decided to further evaluate *FGFR4* expression in 23 primary human liver tumors and 11 normal livers using quantitative real-time polymerase chain reaction (qRT-PCR). The expression of *FGFR4* in each sample was normalized to the expression of this gene in the first normal liver sample (N1). The average level of *FGFR4* expression was moderately increased in liver tumors (1.22-±0.05-fold) compared to normal livers (0.90-±0.04-fold), but the difference did not reach statistical significance (p = 0.23) when that population was considered as a whole (Fig. 6C). However, *FGFR4* expression was significantly higher (more than 2-fold) in a subset of tumors (7/23; 30%). These results illustrate that FGFR4 expression is deregulated in several types of cancer. The increased expression of *FGFR4* in a subset of liver tumors suggests that it may represent an attractive target for the treatment of liver cancer in a diagnostic-selected patient population.

## Discussion

In this study, we provide evidence that FGFR4 participates in hepatocellular carcinoma and that treatment with an FGFR4 inactivating antibody can provide anti-tumor benefits. To evaluate the participation of FGFR4 in liver tumorigenesis we used genetically engineered mouse models. The exogenous expression of FGF19 was shown to promote hepatocyte proliferation, hepatocellular dysplasia, and the development of HCC in mice. In addition, we and others have demonstrated that Klotho β is required for the liver-specific activities of FGF19 [16], [19], [24]. Because KLB and FGFR4 are most highly expressed in liver, we hypothesized that the deregulation of the FGFR4 pathway is responsible for the FGF19-mediated liver tumorigenesis. To test this hypothesis we bred FGF19-TG mice with FGFR4-KO mice. Preneoplastic hepatocellular proliferation and hepatocellular neoplasia were found only in FGF19-TG mice with an FGFR4-WT background. The liver tumorigenesis was abrogated in the FGFR4-KO mice. We further challenged the mice by administering a potent hepatocarcinogen, diethylnitrosamine. Treatment with DEN accelerated the development of HCC in FGF19-TG mice with an FGFR4-WT background, whereas no evidence of liver neoplasia was found in the FGFR4-KO mice. The clear conclusion is that FGFR4 is required for FGF19-promoted liver tumorigenesis.

Together these data suggest a link between FGFR4, liver tumorigenesis, and liver cancer progression. Consequently, FGFR4 is a potential therapeutic target and its inhibition may provide a therapeutic benefit to liver cancer patients. To this end we developed an anti-FGFR4 neutralizing antibody (LD1). LD1 binds to FGFR4 and inhibits ligand binding, pathway activation, regulation of gene expression, cell proliferation, and colony formation in vitro. The site at which LD1 binds to FGFR4 was localized by evaluating the interaction of LD1 with FGFR4 constructs bearing point mutations at sites that are similar between the FGFR4 orthologs but dissimilar in the FGFR1-3 orthologs; these amino acid residues in FGFR4 were substituted with the amino acid residues present at the equivalent positions in FGFR3. LD1 bound to wild type FGFR4 and most of the mutant FGFR4 constructs with the exception of the G165A mutant. The replacement of a glycine by an alanine at position 165 of FGFR4 nearly abolished LD1 binding. The exquisite specificity of LD1 for FGFR4 combined with the high identity of this region between FGFRs emphasizes the importance of this residue for LD1 binding. Glycine 165 in FGFR4 corresponds to alanine 171 in FGFR1. Interestingly, alanine 171 is the residue at the closest approach in the FGFR1 dimer interface [25]. Across the axis of the dimer, the side chain of alanine 171 of one receptor makes a hydrophobic contact with alanine 171 of the adjacent receptor. The sequence conservation in this region of the FGFRs is consistent with this region forming a receptor-receptor interface [25]. Thus, the binding of LD1 to this equivalent region of FGFR4 is likely disrupting receptor dimerization. Ligand-induced receptor dimerization is essential for the activation of FGFRs [26], [27]. Therefore, inhibition of FGFR4 dimerization is a potential mechanism of action for LD1. A similar mechanism of action has already been described for other therapeutic antibodies [28].

We showed that in vivo, LD1 acts on liver cancer xenograft tumors by inhibiting the modulation of genes downstream from FGFR4 and by blocking tumor growth. In addition, the administration of LD1 inhibited the formation and development of HCCs in FGF19-TG mice.

These data demonstrate that FGFR4 is involved in promoting tumorigenesis and cancer progression. In particular, our results suggest that FGFR4 may play an important role in hepatocellular carcinoma. Several lines of evidence support this hypothesis. FGFR4 is the predominant FGFR isoform present in human hepatocytes [15]. We have previously reported that liver tissue has the highest *FGFR4* and *KLB* transcript levels, and both of these proteins are essential for ligand-stimulated activity by this signaling complex [16]. Furthermore, ectopic expression of FGF19 (i.e. FGFR4-specific ligand) in mice promotes hepatocyte proliferation, hepatocellular dysplasia, and neoplasia [18] and FGF19-induced hepatocyte proliferation has been reported to be uniquely mediated by FGFR4 [24]. A recent report suggests that FGFR4 also contributes significantly to HCC progression by modulating alpha-fetoprotein secretion, proliferation, and anti-apoptosis [17]. FGFR4 expression has also been shown to promote resistance to chemotherapy [29]. It should be noted that one group has reported a protective role, rather than an HCC promoting effect, for FGFR4 in mice [30]. It is possible that contextual factors including the identity and concentration of ligand, as well as the levels of FGFRs and co-receptor expression might modulate the role of FGFR4 in tumorigenesis. For example, we found *FGFR4* expression to be significantly increased in a subset of primary liver tumors, suggesting that FGFR4 may represent an attractive target for the treatment of liver cancer in a diagnostic-selected patient population. Given the accumulating evidence for the participation of FGFR4 in liver tumorigenesis and HCC progression, we believe that a therapeutic intervention that includes an anti-FGFR4 neutralizing antibody is likely to be beneficial in the treatment of liver cancer.

## Materials and Methods

### Ethics Statement

All animal studies were conducted in accordance with the Guide for the Care and Use of Laboratory Animals, published by the National Institutes of Health (NIH) (NIH Publication 8523, revised 1985). The Institutional Animal Care and Use Committee (IACUC) at Genentech reviewed and approved all animal protocols. The approval IDs for this study are: 02–258, 04–0161, 06–1387 A, 06–1581 I, 07–0978 B, and 07–0978 C.

### In silico Expression Analysis

For expression analysis, box- and whisker-plots were generated for *FGFR4* with the normalized gene expression data extracted from the BioExpress™ database (Gene Logic, Gaithersburg, MD). The distribution of *FGFR4* expression in normal and cancer tissues was evaluated using the signals associated with probe 204579_at.

### Immunohistochemistry

Formalin-fixed paraffin-embedded tissue sections were treated for antigen retrieval using Trilogy (Cell Marque, Rocklin, CA) and then incubated with 10 µg/mL anti-FGFR4 antibody (8G11; Genentech, South San Francisco, CA). The immunostaining was accomplished using a biotinylated secondary antibody, an ABC-HRP reagent (Vector Laboratories, Burlingame, CA), and a metal-enhanced DAB colorimetric peroxidase substrate (Thermo Fisher Scientific, Rockford, IL).

### Semi-quantitative RT-PCR

Total RNA was extracted using the RNeasy kit (Qiagen, Valencia, CA). Specific primers and fluorogenic probes were used to amplify and quantitate gene expression [31]. The gene-specific signals were normalized to the *RPL19* housekeeping gene. All TaqMan qRT-PCR reagents were purchased from Applied Biosystems (Foster City, CA). A minimum of a triplicate set of data was analyzed for each condition. Data are presented as the mean ± SEM.

### Immunoprecipitation and Immunoblotting

Lysates of cultured cells or frozen tissues were prepared with RIPA lysis buffer (Millipore, Billerica, MA) supplemented with Complete EDTA-free protease inhibitor cocktail (Roche, Indianapolis, IN), phosphatase inhibitor cocktails 1 and 2 (Sigma-Aldrich, St. Louis, MO), 2 mM sodium fluoride, and 2 mM sodium orthovanadate. Equal amounts of protein, as determined by BCA assay (Thermo Fisher Scientific) were analyzed by immunoblot analysis using antibodies against FGFR4 (8G11; Genentech), FGFR3 (Santa Cruz Biotechnology, Santa Cruz, CA), FGFR2 (GeneTex, Irvine, CA) and FGFR1 (Santa Cruz Biotechnology). For the human liver lysates, the immunoblot analysis was preceded by the immunoprecipitation of FGFR4 as described previously [16].

### Generation of FGFR4 Monoclonal Antibodies

*FGFR4* null mutant (i.e. FGFR4-KO) mice were immunized with recombinant human and mouse FGFR4-Fc chimeric proteins (Genentech). Spleens were harvested after 8 weeks and hybridomas were generated. Cultured supernatants were collected and screened by solid phase antibody binding assay against the immunogens. Positive cell lines were further screened using solid phase receptor binding assay for their efficacy at inhibiting FGF1 and FGF19 binding to human and mouse FGFR4. The LD1-producing hybridoma was subcloned twice to insure monoclonality.

### Molecular Cloning of LD1

Total RNA was extracted from hybridoma cells producing muLD1 using the RNeasy Mini kit (Qiagen). The variable light and variable heavy domains were amplified using reverse transcription-PCR (RT-PCR). The forward primers were specific for the NH_2_-terminal amino acid sequence of the variable light and variable heavy regions. Respectively, the light chain and heavy chain reverse primers were designed to anneal to regions in the constant light and constant heavy domain 1 that are highly conserved across species. Amplified variable light chain was cloned into a mammalian expression vector containing the human κ constant domain. Amplified variable heavy chain was inserted into a mammalian expression vector encoding the full-length human IgG1 constant domain. The chimeric antibody was transiently expressed as previously described [16].

### Solid Phase Antibody Binding Assay

Maxisorp 96 well plates were coated overnight at 4°C with 50 µL of 2 µg/mL anti-human immunoglobulin Fc fragment-specific (Jackson ImmunoResearch Laboratories, West Grove, PA) or anti-FLAG antibody (Sigma-Aldrich). The non-specific binding sites were saturated with 200 µL PBS/3% bovine serum albumin (BSA) for 1 hour and FGFRs-IgG (Genentech and R&D Systems, Minneapolis, MN) or FLAG tagged-FGFR4 (FGFR4ΔTM-FLAG) were incubated in PBS/0.3% BSA for 1 hour. The plates were washed and incubated for 1 hour with anti-FGFR4 antibodies in PBS/0.3% BSA. The bound antibodies were detected using an HRP-conjugated anti-IgG (Jackson ImmunoResearch Laboratories) and the TMB peroxidase colorigenic substrate (KPL, Gaithersburg, MD).

### Flow Cytometry Analysis

Cells for flow cytometry analysis were resuspended with PBS containing 5 mM EDTA and washed with PBS containing 2% heat-inactivated fetal bovine serum (FBS). All subsequent steps were carried out on ice. Cells (1×10^6^) were incubated with a primary antibody (LD1 or isotype control) for 30 minutes, followed by incubation with phycoerythrin (PE)-conjugated anti-human IgG antibody (Jackson ImmunoResearch). Cells were analyzed with a FACScan flow cytometer (BD Biosciences, San Jose, CA).

### DNA Constructs

The human FGFR4 (hFGFR4) cDNA was cloned as described previously [16]. The extracellular domain of FGFR4 was also subcloned into the expression vector pCMV-Tag4A (Stratagene, La Jolla, CA) to obtain a secreted form of FGFR4 with a FLAG tag at the C-terminal end (FGFR4ΔTM-Flag). Single nucleotide mutations were introduced in the FGFR4ΔTM-Flag constructs using the QuikChange XL Site-Directed Mutagenesis Kit (Stratagene). We also generated a human FGFR4-FGFR1 chimeric construct (hFGFR4/R1) that contained the extracellular and the transmembrane domains of human FGFR4 fused to the cytoplasmic domain of human FGFR1. The amino acid sequence joining FGFR4 (bold) to FGFR1 (plain) is ···**AVLLLLAGLYRG**KMKSG···. The hFGFR4 cDNA or hFGFR4/R1 cDNA was ligated into the pQCXIP retroviral bicistronic expression vector (Clontech Laboratories, Mountain View, CA).

### FGFR4ΔTM-Flag-conditioned Medium

HEK293 cells were transfected with the wild type or mutant FGFR4ΔTM-Flag constructs or the corresponding empty vector and maintained in serum free PS25 medium for 72 to 96 hours. The resulting media were filtered, supplemented with HEPES pH 7.2 (final concentration 40 mM), and protease inhibitors and stored at 4°C until used.

### Cell Culture and Stable Cell Lines

HEK293, HEPG2, and HEP3B cells were obtained from American Type Culture Collection (ATCC, Manassas, VA) and maintained in F-12:DMEM mix (50∶50) supplemented with 10% FBS and 2 mM L-glutamine. HUH7 and PLC/PRF/5 cells were cultured in DMEM high glucose, 10% FBS. JHH4, JHH5, and JHH7 cells were purchased from the Japanese Cancer Research Resources Bank (Tokyo, Japan) and maintained in Williams Medium E supplemented with 10% FBS and 2 mM L-glutamine. SNU449 cells were obtained from ATCC and maintained in RPMI 1640 containing 10% FBS and 2 mM L-glutamine. BaF3 cells were maintained in RPMI 1640 (Life Technologies, Carlsbad, CA) supplemented with 10% FBS, 1 ng/mL IL-3, and 2 mM L-glutamine. L6 cells were obtained from ATCC and maintained in DMEM high glucose supplemented with 10% FBS.

Cultures of BaF3 and L6 cells were infected with the empty, hFGFR4, or hFGFR4/R1 retroviral expression vectors according to the manufacturer’s recommendations and selected in media containing 2.5 µg/mL puromycin (Life Technologies) for 10 to 12 days. From the selected pools, the top five percent of highest expressing cells was isolated by Fluorescence Activated Cell Sorting (FACS) using an anti-FGFR4 antibody (8G11; Genentech). The resulting pools of cells expressing high levels of FGFR4, high levels of FGFR4/R1, and the control cells stably transfected with an empty vector were maintained in complete medium containing 2.5 µg/mL puromycin.

### Mitogenic Assays

BaF3/control, BaF3/FGFR4, and BaF3/FGFR4/R1 cells were washed twice and seeded in 96-well plates (22,500 cells/well) in RPMI 1640 supplemented with 10% FBS, 2 mM L-glutamine, and 2 µg/mL heparin. FGFs were added to each well and the cells were incubated at 37°C for 72 hours. The relative cell density was measured using CellTiter-Glo (Promega, Madison, WI) according to the manufacturer’s recommendations.

### Anti-FGFR4 Antibody Inhibition of FGF Pathway Activation

Cells were serum starved for 24 hours in the absence or presence of LD1 or an isotype control antibody. They were then stimulated with 5 ng/mL FGF1 (FGF acidic, R&D Systems) and 10 µg/mL heparin for 5 minutes. The cells were lysed with RIPA lysis buffer (Millipore) supplemented with Complete EDTA-free protease inhibitor cocktail (Roche), phosphatase inhibitor cocktails 1 and 2 (Sigma-Aldrich), 2 mM sodium fluoride, and 2 mM sodium orthovanadate. Equal amounts of protein were analyzed by immunoblot using antibodies against phospho-ERK1/2, phospho-FRS2, ERK1/2 (Cell Signaling Technology, Danvers, MA), and FRS2 (Millipore).

### Clonogenic Assay

HUH7 (5,000 cells/well), PLC/PRF/5 (2,000 cells/well), JHH5 (500 cells/well), or JHH5/hFGFR4 shRNA (500 cells/well) cells were seeded in 2 mL medium/well in 6 well plates, in triplicate. Three hours after seeding, the HUH7 and PLC/PRF/5 cells were treated without or with anti-FGFR4 antibody (chLD1; Genentech). Antibody was replaced twice weekly for the duration of the experiment (14 days). For the JHH5 and JHH5/hFGFR4 shRNA cells, treatment without or with 2 µg/mL doxycycline was initiated 3 hours after seeding, and replaced three times weekly for the duration of the experiment. Cells were washed with PBS and stained with 0.5% crystal violet solution. Colonies were counted using MetaMorph software (Molecular Devices, Sunnyvale, CA).

### In vivo Experiments

Female FVB mice that were 5 to 6 weeks old were obtained from Charles River Laboratories International (Wilmington, MA). Mice were given intraperitoneal (IP) injections (10 mg/kg) of a control or an anti-FGFR4 (chLD1) antibody. Forty-eight hours later, the mice received vehicle (PBS) or 1 mg/kg FGF19 intravenously (IV); the mice were provided standard feed and water *ad libitum* until 12 hours before injection with FGF19, at which time feed was removed. After 30 minutes, mice from all groups were necropsied and tissue samples were collected, frozen in liquid nitrogen, and stored at −70°C. Total RNA from frozen tissue samples was prepared using the RNeasy kit (Qiagen). Groups of 3 to 5 animals were analyzed for each condition. Data are presented as the mean ± SEM and were analyzed by the Student’s t-test.

For xenograft experiments, 6- to 8-week-old nu/nu female mice (Charles River Laboratories International) were inoculated subcutaneously with 5×10^6^ cells (200 µL/mouse) and Matrigel (BD Biosciences). Mice bearing tumors of equivalent volumes (∼150 mm^3^) were randomized into groups (n = 10) and treated IP once weekly in the initial experiment and twice weekly in the repeat experiment. Tumors were measured with an electronic caliper (Fowler Sylvac Ultra-Cal Mark III; Fred V. Fowler Company, Newton, MA) and average tumor volume was calculated using the formula: (W^2^×L)/2 where W and L are the smaller diameter and larger diameter, respectively. Data are presented as the mean tumor volume ± SEM and were analyzed by the Student’s t-test.

The *FGF19* transgenic mice were produced as described previously [32]. The FGFR4-KO animals were constructed as reported previously [33] and provided by W. L. McKeehan (University of Texas Southwestern Medical Center, Dallas, TX). Mice that both overexpressed FGF19 and lacked the FGFR4 receptor (FGF19-TG:FGFR4-KO) were fabricated by crossing young adult FGF19-TG males with young adult FGFR4-KO females. The presence of both gene engineering events was confirmed at weaning by PCR on tail DNA.

## Supporting Information

Figure S1 **LD1 binds to FGFR4.** *A*, LD1 binds to HEK293 cells transiently transfected with a human (*hFGFR4*) expression construct, but not to HEK293 cells transfected with control empty vector. *B*, LD1 binds to HEK293 cells transiently transfected with an *hFGFR4* expression construct, but not to HEK293 cells transfected with *hFGFR1*, *hFGFR2*, or *hFGFR3* expression constructs. *C*, LD1 binds to JHH5 cells endogenously expressing FGFR4. JHH5 cells stably transfected with *FGFR4* shRNA exhibit diminished LD1 binding upon treatment with 2 µg/mL doxycycline for 48 and 96 hours.
(TIF)

Figure S2 **Expression of FGFRs in liver cancer cell lines.** *A*, *FGFR1-FGFR4* mRNA expression in a panel of liver tumor cell lines as determined by qRT-PCR. The values are represented as fold expression relative to the *FGFR1* levels in the JHH4 cell line. *B*, FGFR4 protein expression in the same panel of cell lines as in Fig. S2A as determined by Western blot.
(TIF)

Figure S3 **LD1 inhibits FGFR4 biological activities in HUH7 cells.** LD1 inhibits the FGFR4-regulated *CYP7A1* repression in HUH7 cells. *CYP7A1* levels are represented as fold expression relative to the level in untreated cells.
(TIF)

Figure S4 **In vivo efficacy of LD1.** LD1 (30 mg/kg; twice weekly) inhibits HUH7 xenograft tumor growth in vivo. The anti-tumor efficacy of LD1 was evaluated in a biweekly modality.
(TIF)

Table S1 **FGFR4 expression in normal and cancer tissues.** Prevalence of FGFR4 expression in normal and cancer tissues as determined by histopathological evaluation of FGFR4 immunostaining.
(TIF)

## References

[1] Ornitz DM, Itoh N (2001). Fibroblast growth factors.. Genome Biol.

[2] Eswarakumar VP, Lax I, Schlessinger J (2005). Cellular signaling by fibroblast growth factor receptors.. Cytokine Growth Factor Rev.

[3] Powers CJ, McLeskey SW, Wellstein A (2000). Fibroblast growth factors, their receptors and signaling.. Endocr Relat Cancer.

[4] Bange J, Prechtl D, Cheburkin Y, Specht K, Harbeck N (2002). Cancer progression and tumor cell motility are associated with the FGFR4 Arg(388) allele.. Cancer Res.

[5] Cappellen D, De Oliveira C, Ricol D, de Medina S, Bourdin J (1999). Frequent activating mutations of FGFR3 in human bladder and cervix carcinomas.. Nat Genet.

[6] Chesi M, Brents LA, Ely SA, Bais C, Robbiani DF (2001). Activated fibroblast growth factor receptor 3 is an oncogene that contributes to tumor progression in multiple myeloma.. Blood.

[7] Chesi M, Nardini E, Brents LA, Schrock E, Ried T (1997). Frequent translocation t(4;14)(p16.3;q32.3) in multiple myeloma is associated with increased expression and activating mutations of fibroblast growth factor receptor 3.. Nat Genet.

[8] Gowardhan B, Douglas DA, Mathers ME, McKie AB, McCracken SR (2005). Evaluation of the fibroblast growth factor system as a potential target for therapy in human prostate cancer.. Br J Cancer.

[9] Jaakkola S, Salmikangas P, Nylund S, Partanen J, Armstrong E (1993). Amplification of fgfr4 gene in human breast and gynecological cancers.. Int J Cancer.

[10] Jang JH, Shin KH, Park JG (2001). Mutations in fibroblast growth factor receptor 2 and fibroblast growth factor receptor 3 genes associated with human gastric and colorectal cancers.. Cancer Res.

[11] Jang JH, Shin KH, Park YJ, Lee RJ, McKeehan WL (2000). Novel transcripts of fibroblast growth factor receptor 3 reveal aberrant splicing and activation of cryptic splice sequences in colorectal cancer.. Cancer Res.

[12] Jeffers M, LaRochelle WJ, Lichenstein HS (2002). Fibroblast growth factors in cancer: therapeutic possibilities.. Expert Opin Ther Targets.

[13] Xiao S, Nalabolu SR, Aster JC, Ma J, Abruzzo L (1998). FGFR1 is fused with a novel zinc-finger gene, ZNF198, in the t(8;13) leukaemia/lymphoma syndrome.. Nat Genet.

[14] Shariff MI, Cox IJ, Gomaa AI, Khan SA, Gedroyc W (2009). Hepatocellular carcinoma: current trends in worldwide epidemiology, risk factors, diagnosis and therapeutics.. Expert Rev Gastroenterol Hepatol.

[15] Kan M, Wu X, Wang F, McKeehan WL (1999). Specificity for fibroblast growth factors determined by heparan sulfate in a binary complex with the receptor kinase.. J Biol Chem.

[16] Lin BC, Wang M, Blackmore C, Desnoyers LR (2007). Liver-specific activities of FGF19 require Klotho beta.. J Biol Chem.

[17] Ho HK, Pok S, Streit S, Ruhe JE, Hart S (2009). Fibroblast growth factor receptor 4 regulates proliferation, anti-apoptosis and alpha-fetoprotein secretion during hepatocellular carcinoma progression and represents a potential target for therapeutic intervention.. J Hepatol.

[18] Nicholes K, Guillet S, Tomlinson E, Hillan K, Wright B (2002). A mouse model of hepatocellular carcinoma : ectopic expression of fibroblast growth factor 19 in skeletal muscle of transgenic mice.. Am J Pathol.

[19] Kurosu H, Choi M, Ogawa Y, Dickson AS, Goetz R (2007). Tissue-specific expression of betaKlotho and fibroblast growth factor (FGF) receptor isoforms determines metabolic activity of FGF19 and FGF21.. J Biol Chem.

[20] Pai R, Dunlap D, Qing J, Mohtashemi I, Hotzel K (2008). Inhibition of fibroblast growth factor 19 reduces tumor growth by modulating beta-catenin signaling.. Cancer Res.

[21] Ornitz DM, Yayon A, Flanagan JG, Svahn CM, Levi E (1992). Heparin is required for cell-free binding of basic fibroblast growth factor to a soluble receptor and for mitogenesis in whole cells.. Mol Cell Biol.

[22] Holt JA, Luo G, Billin AN, Bisi J, McNeill YY (2003). Definition of a novel growth factor-dependent signal cascade for the suppression of bile acid biosynthesis.. Genes Dev.

[23] Desnoyers LR, Pai R, Ferrando RE, Hotzel K, Le T (2008). Targeting FGF19 inhibits tumor growth in colon cancer xenograft and FGF19 transgenic hepatocellular carcinoma models.. Oncogene.

[24] Wu X, Ge H, Gupte J, Weiszmann J, Shimamoto G (2007). Co-receptor requirements for fibroblast growth factor-19 signaling.. J Biol Chem.

[25] Plotnikov AN, Schlessinger J, Hubbard SR, Mohammadi M (1999). Structural basis for FGF receptor dimerization and activation.. Cell.

[26] Ibrahimi OA, Yeh BK, Eliseenkova AV, Zhang F, Olsen SK (2005). Analysis of mutations in fibroblast growth factor (FGF) and a pathogenic mutation in FGF receptor (FGFR) provides direct evidence for the symmetric two-end model for FGFR dimerization.. Mol Cell Biol.

[27] Mohammadi M, Olsen SK, Ibrahimi OA (2005). Structural basis for fibroblast growth factor receptor activation.. Cytokine Growth Factor Rev.

[28] Agus DB, Akita RW, Fox WD, Lewis GD, Higgins B (2002). Targeting ligand-activated ErbB2 signaling inhibits breast and prostate tumor growth.. Cancer Cell.

[29] Roidl A, Berger HJ, Kumar S, Bange J, Knyazev P (2009). Resistance to chemotherapy is associated with fibroblast growth factor receptor 4 up-regulation.. Clin Cancer Res.

[30] Huang X, Yang C, Jin C, Luo Y, Wang F (2009). Resident hepatocyte fibroblast growth factor receptor 4 limits hepatocarcinogenesis.. Mol Carcinog.

[31] Winer J, Jung CK, Shackel I, Williams PM (1999). Development and validation of real-time quantitative reverse transcriptase-polymerase chain reaction for monitoring gene expression in cardiac myocytes in vitro.. Anal Biochem.

[32] Tomlinson E, Fu L, John L, Hultgren B, Huang X (2002). Transgenic mice expressing human fibroblast growth factor-19 display increased metabolic rate and decreased adiposity.. Endocrinology.

[33] Yu C, Wang F, Kan M, Jin C, Jones RB (2000). Elevated cholesterol metabolism and bile acid synthesis in mice lacking membrane tyrosine kinase receptor FGFR4.. J Biol Chem.

