# Supplementary figures and images for: Targeting FGFR4 Inhibits Hepatocellular Carcinoma in Preclinical Mouse Models
Source: PLoS One. 2012 May 15;7(5):e36713. doi: 10.1371/journal.pone.0036713 (PMC3352934; doi:10.1371/journal.pone.0036713)

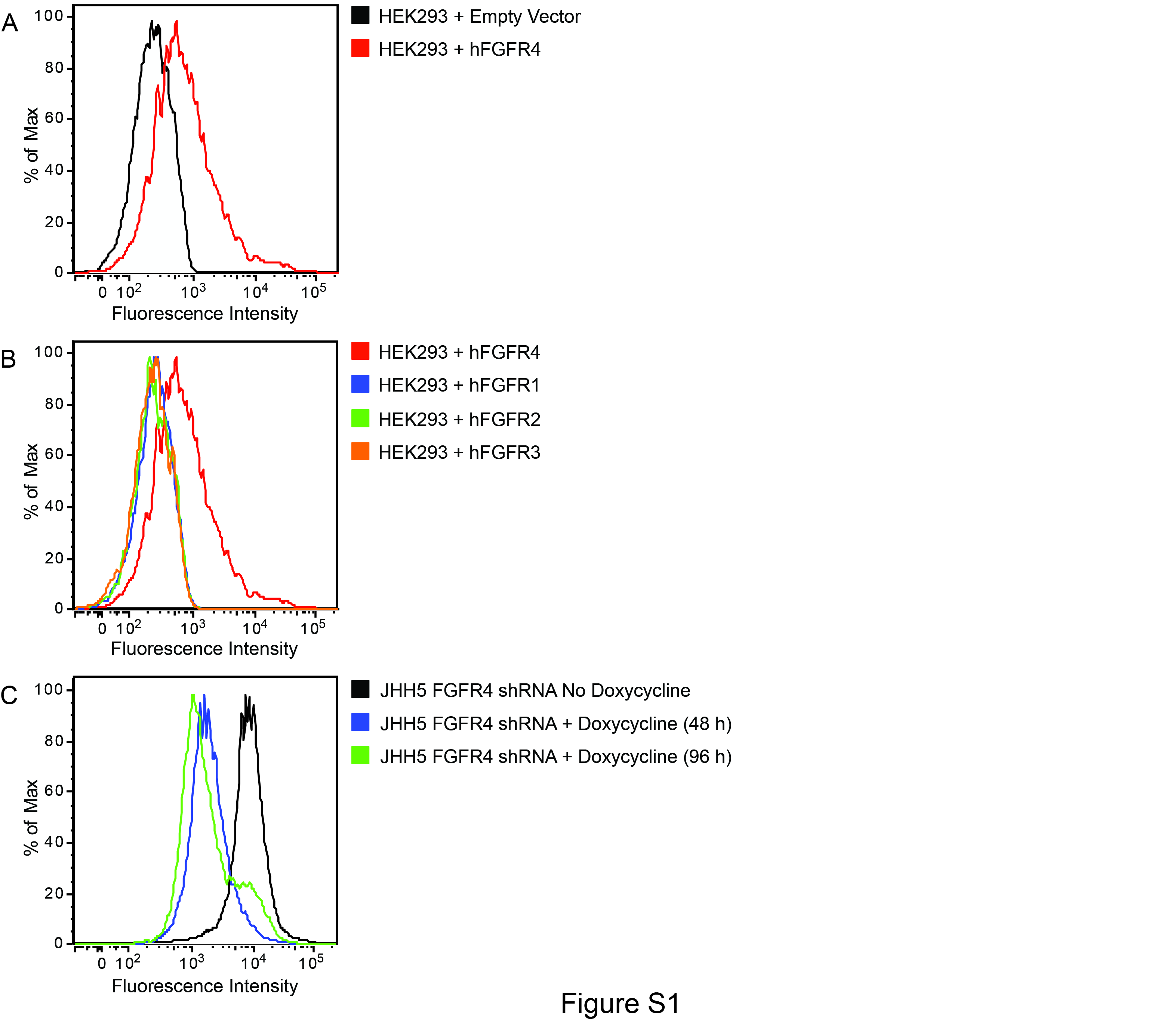

Supplement: Figure S1 — LD1 binds to FGFR4. A, LD1 binds to HEK293 cells transiently transfected with a human (hFGFR4) expression construct, but not to HEK293 cells transfected with control empty vector. B, LD1 binds to HEK293 cells transiently transfected with an hFGFR4 expression construct, but not to HEK293 cells transfected with hFGFR1, hFGFR2, or hFGFR3 expression constructs. C, LD1 binds to JHH5 cells endogenously expressing FGFR4. JHH5 cells stably transfected with FGFR4 shRNA exhibit diminished LD1 binding upon treatment with 2 µg/mL doxycycline for 48 and 96 hours. (TIF) [file pone.0036713.s001.tif]

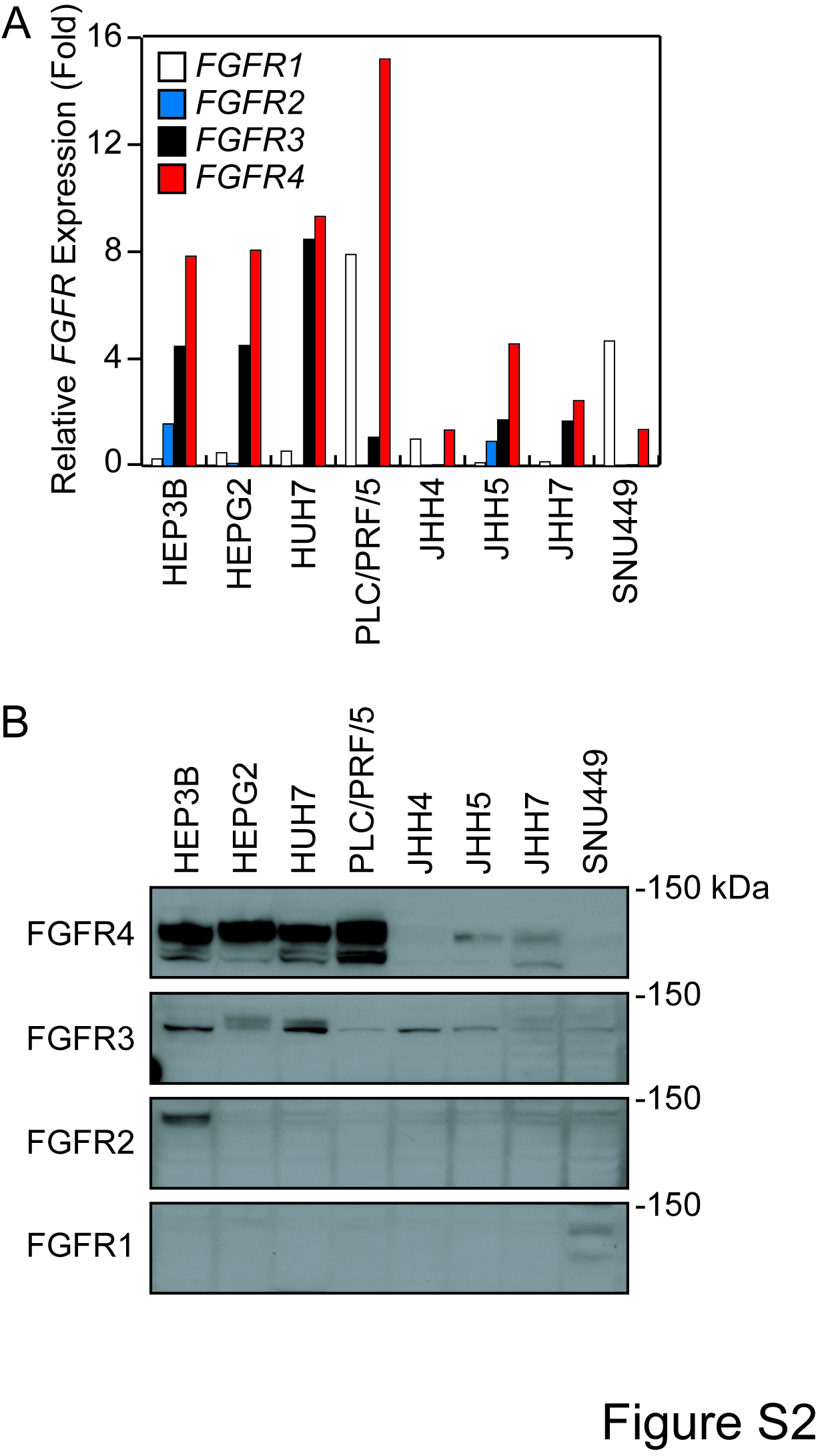

Supplement: Figure S2 — Expression of FGFRs in liver cancer cell lines. A, FGFR1-FGFR4 mRNA expression in a panel of liver tumor cell lines as determined by qRT-PCR. The values are represented as fold expression relative to the FGFR1 levels in the JHH4 cell line. B, FGFR4 protein expression in the same panel of cell lines as in Fig. S2A as determined by Western blot. (TIF) [file pone.0036713.s002.tif]

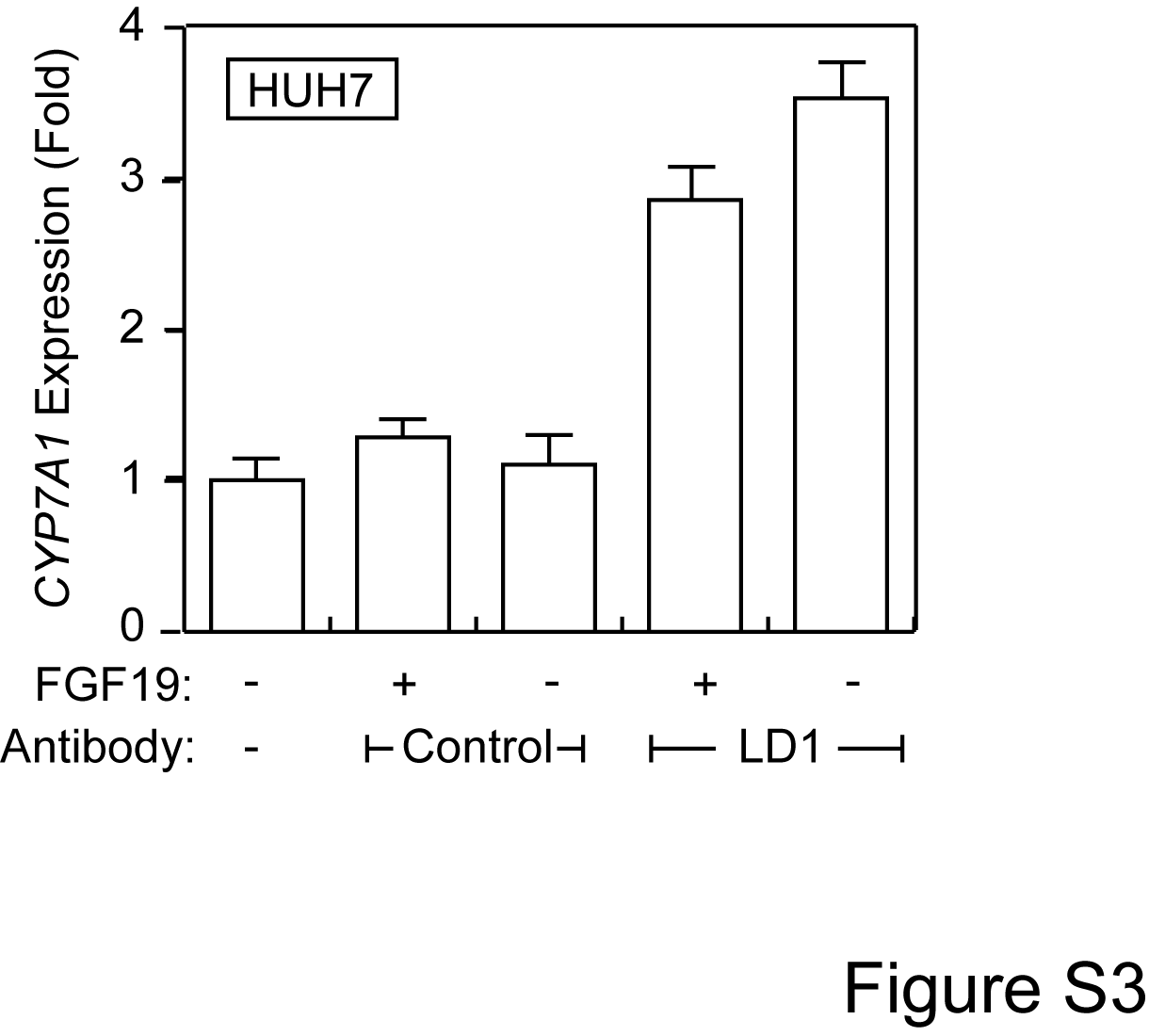

Supplement: Figure S3 — LD1 inhibits FGFR4 biological activities in HUH7 cells. LD1 inhibits the FGFR4-regulated CYP7A1 repression in HUH7 cells. CYP7A1 levels are represented as fold expression relative to the level in untreated cells. (TIF) [file pone.0036713.s003.tif]

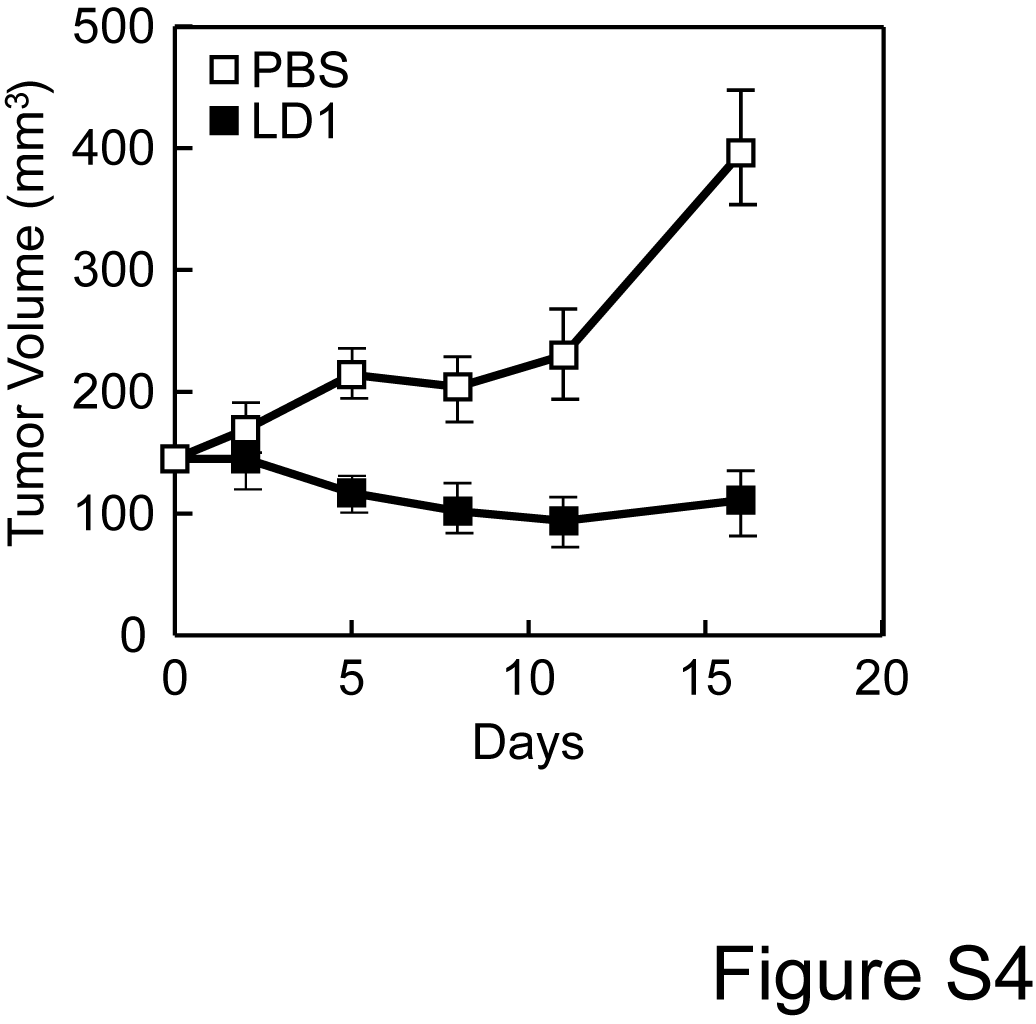

Supplement: Figure S4 — In vivo efficacy of LD1. LD1 (30 mg/kg; twice weekly) inhibits HUH7 xenograft tumor growth in vivo. The anti-tumor efficacy of LD1 was evaluated in a biweekly modality. (TIF) [file pone.0036713.s004.tif]

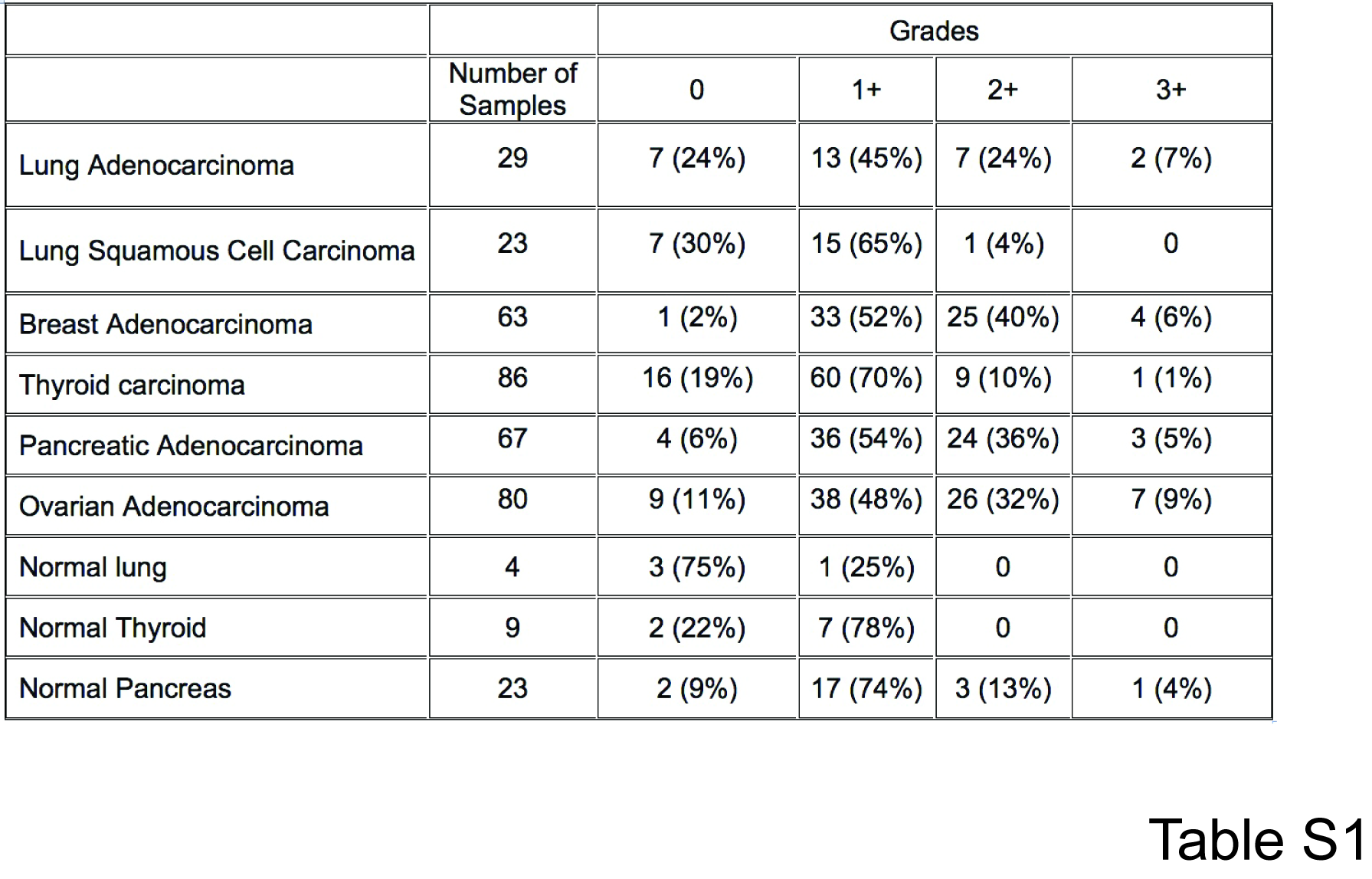

Supplement: Table S1 — FGFR4 expression in normal and cancer tissues. Prevalence of FGFR4 expression in normal and cancer tissues as determined by histopathological evaluation of FGFR4 immunostaining. (TIF) [file pone.0036713.s005.tif]
